# Explanatory

**Published:** 1889-08

**Authors:** 


### Explanatory.

On another page of this number of the REGISTER is given an explanatory statement from the American Dental Trade Association. We publish this that the profession may have a statement from the association itself of its plan of operation, its aims, objects, etc.

If this statement is full and accurate, there is very little ground for the severe criticism and remark that has so often been indulged in regard to this organization. It seems quite reasonable to accept this statement as correct, until good evidence to the contrary is presented; this we think in all fairness will be granted to every one.

The mere fact of association of merchants and trades people does not necessarily involve any thing wrong; they may be used for improper purposes, and so may any organization, but if rightly used, they may be the means of great good in controlling and holding in check unjust and pernicious competition and irregularities in trade; it may also be used for securing better products in manufacture. As to whether these results are now attained each one must judge for himself. It is beyond question that dentists are to-day better supplied with needed material and facilities for the practice of the profession than ever before, and almost every appliance and product is better, and at as low a cost as good material has ever been afforded heretofore.

While dentists should not submit to exorbitant charges, it should be borne in mind that there is something of vastly greater importance than the low cost of supplies, and that is the quality of these supplies. The difference between an instrument of an excellent and durable quality and one of poor quality can hardly be estimated by dollars and cents. We have not one word of extenuation for any wrong-doing that this association may indulge, but it is hardly just or proper to denounce in advance or in the absence of evidence. Any organization of men may be used for bad purposes, as a great many excellent ones have been, but we are not therefore always to condemn the association.

We have all along sought to keep advised as to the working of this trade association and have always been ready to commend it in well-doing, and to condemn any crookedness that might appear in its working.

